# Effectiveness of three different types of educational methods on implementation of proper oral hygiene behaviour prior to orthodontic treatment

**DOI:** 10.1590/2177-6709.26.1.e2119248.oar

**Published:** 2021-03-22

**Authors:** Fethiye CAKMAK OZLU, Erol AKTUNC, Hakan YILMAZ, Ersan Ilsay KARADENIZ

**Affiliations:** 1 Department of Orthodontics, Faculty of Dentistry, Ondokuz Mayıs University (Samsun, Turkey).; 2 Department of Family Medicine, Faculty of Medicine, Bülent Ecevit University (Zonguldak, Turkey).; 3 Department of Orthodontics, Faculty of Dentistry, Yeditepe University (Istanbul, Turkey).; 4 Department of Orthodontics, College of Medicine and Dentistry, James Cook University (Cairns, QLD, Australia).

**Keywords:** Orthodontic appliances, Oral hygiene, Oral health training

## Abstract

**Objective:**

The aim of this study was to compare three teaching methods’ time and personnel requirements, and their effects on plaque and gingival indices.

**Methods:**

This study was a single-blind randomized controlled trial on fixed orthodontic appliance candidates (n = 90), assigned into a control group (n = 30) and two different study groups (n = 30 each). The control group received standard printed educational material and was assisted with verbal information. The study groups either received video-assisted or hands-on training about fixed orthodontic appliance and oral hygiene. The time requirements for all three educational interventions was recorded during the initial visit. The adequacy of oral hygiene was documented through plaque and gingival indices during the initial visit and eighth week of the treatment. The continuous variables were analyzed using 1-way ANOVA. Tukey HSD and Student *t*-tests were used for *post-hoc* comparisons (α?#8197;= 0.05). Also, a chi-square test was used for the analysis of categorical variables.

**Results:**

Standard education failed to maintain the plaque and gingival indices at the eighth week of the treatment. Although both video-assisted and hands-on training took a considerable amount of time, they served well in preserving both of the indices at the eighth week. The longer the educational intervention was, the better the preservation of the plaque and gingival indices.

**Conclusion:**

Educational intervention, either with video-assisted or hands-on programs, provided better results in oral hygiene depending on the time and personnel constraints of the orthodontist.

## RESUMO

**Objetivo:**

O objetivo do presente estudo foi comparar a duração e os pré-requisitos de equipe de três métodos de orientação ao paciente, e seus efeitos sobre os índices de placa e de sangramento gengival.

**Métodos:**

Esse foi um estudo controlado aleatório cego em candidatos (n=90) a tratamento ortodôntico fixo, que foram divididos em um grupo controle (n=30) e dois grupos de estudo (n= 30 cada). O grupo controle recebeu material educacional impresso e foi orientado com informações verbais. Os grupos de estudo receberam treinamento em formato de vídeo ou treinamento *hands-on* sobre o tratamento com Ortodontia fixa e higiene bucal. O tempo necessário para cada um dos três tipos de treinamento foi registrado durante a visita inicial. A qualidade da higiene bucal foi avaliada por meio dos índices de placa e de sangramento gengival durante a visita inicial e na oitava semana de tratamento. As variáveis contínuas foram analisadas utilizando-se *one-way* ANOVA. Os testes HSD de Tukey e *t* de Student foram utilizados para comparações *post-hoc* (α?#8197;= 0,05), e o teste qui-quadrado foi utilizado para análise das variáveis categóricas.

**Resultados:**

O método de ensino convencional fracassou em manter os índices de placa e de sangramento gengival até a oitava semana de tratamento. Apesar de tanto o método de treinamento com vídeos e de treinamento *hands-on* demandarem um tempo considerável, eles conseguiram manter ambos os índices até a oitava semana. Quanto mais longa a orientação realizada, melhor foi a manutenção dos índices de placa e de sangramento gengival.

**Conclusão:**

Tanto o método de treinamento com vídeo quanto no formato *hands-on* obtiveram melhores resultados de higiene bucal, mas dependem do tempo e das limitações de equipe de cada ortodontista.

## INTRODUCTION

Orthodontic treatment using fixed orthodontic appliances that prevent patients from effectively cleaning their teeth results in increased plaque formation and deterioration of oral hygiene.^1^ Inappropriate oral hygiene behaviour during the fixed orthodontic appliance treatment causes gingival enlargement, gingivitis, enamel decalcification and white spots in the vicinity of the applied therapeutic material in 50-70% of patients with fixed appliances.^1^ ^-^ ^5^ Also, inappropriate oral hygiene has been shown to prolong the treatment duration and may result in poor treatment outcomes.^6^ Of all the orthodontic treatments, 5-10% fail because of inappropriate oral hygiene caused by patient incompliance.^7^ For these reasons, proper oral hygiene behaviour is of great importance to the treatment period of almost two years. Oral hygiene can only be achieved through patient compliance, which is built up through communication between the orthodontist, the patient and the family by means of verbal and written educational material.^8^ Routine oral hygiene instruction given to the patients by the orthodontist may be insufficient to provide proper oral hygiene.^5^ ^,^ ^6^

The implementation of oral hygiene in fixed orthodontic appliance treatment candidates through verbal, written and visual information materials has been evaluated in a couple of studies. They either calculated the ratio of disclosed plaque,^9^ ^-^ ^12^ or plaque and gingival indices^13^ ^-^ ^15^ were objectively used in order to evaluate the implementation of learned information. In these studies, plaque disclosing - one of the methods of oral hygiene motivation - has been shown to increase the patient’s motivation through visualization and presentation.^11^ ^,^ ^12^ However, the messages delivered through text, WhatsApp, etc. indicate that motivation is effective in patients’ oral hygiene motivation,^14^ ^,^ ^16^ and even better than plaque disclosing tablets.^10^ Studies comparing the effectiveness of different oral hygiene training methods found that video-assisted and hands-on training have also been effective approaches.^15^ ^,^ ^17^ In recent reviews, motivational approaches with the potential to make more behavioural changes than traditional health education have been found to have different effects on success rates.^18^ ^,^ ^19^ However, motivation of patients through various methods plays a crucial part in maintaining proper oral hygiene.

In all of these studies, different methods of implementing the proper oral hygiene were evaluated only for their effectiveness. As education is the most important part of building rapport between the clinician and patient, it is also a time and effort-consuming process. Moreover, it may not be as fruitful as expected due to patient compliance problems.^19^ Patient motivation and willingness to persist with lifestyle changes are prone to wear out as they require constant effort on the patients’ behalf to change improper but ingrained habits. The two possible restrictions influencing the transfer of information -either positively or negatively- to any patient are time and personnel requirements in busy orthodontic clinics, which have not been addressed in previous studies. 

Therefore, the aim of this study was to compare the time requirements of three types of information transfer techniques, which were all proven to be effective to different degrees in improving oral hygiene in fixed orthodontic appliance treatment candidates.^18^ ^,^ ^19^ We have also documented the effectiveness rate for each method through plaque and gingival indices. The first null hypothesis was that none of the oral hygiene education methods would affect oral hygiene. The second null hypothesis was that there is no difference in duration of oral hygiene adoption either with video-assisted or hands-on training. 

## MATERIAL AND METHODS

**Figure 1:**
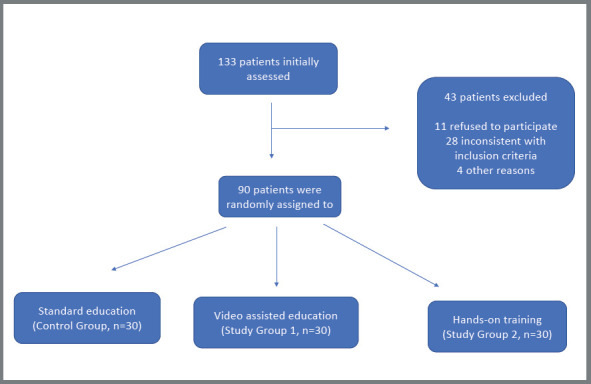
Flow chart of study, selection of patients and arrangement of trial.

This study is a randomized controlled three-arm parallel trial performed on a group of patients who applied to the outpatient clinic of Orthodontics at the Faculty of Dentistry of Bulent Ecevit University on November 11^th^, 2014. We collected a total of 90 patients (mean=14.73±2.63, 10-24 years old) who were undergoing fixed orthodontic treatment after meeting eligibility criteria (Fig 1). According to calculations made with Piface (version 1.76), the minimum sample size that would guarantee power equal to 0.82 was 30 for each of the three groups (α=0.05, SD= 6.61). The study was approved by the ethical committee for human research at the Faculty of Medicine (decision number 2014103, made on 11 February, 2014) at Bulent Ecevit University. All of the participants or their legal representatives gave their informed consent prior to educational intervention. Also, the trial was registered with the ClinicalTrials.gov, supported by the U.S. National Library of Medicine (NCT04018534).

Thirty patients were selected according to the following inclusion criteria: they required non-extraction fixed orthodontic treatment, agreed to use conventional stainless steel brackets (Gemini series, 3M Unitek, Monrovia, California, USA), had at least 20 natural permanent teeth, crowding under 5mm in the incisors, and were willing to participate in this research. The exclusion criteria were as follows: having dental caries, periodontal disease, systemic or chronic diseases such as diabetes, physical or mental disorders, smoking, having undergone previous orthodontic treatment, extensive dental restorations, antibiotic use during the previous three months, dental fluorosis and use of electric toothbrushes. 

This study was conducted as a single-blind, randomized clinical trial. A computer program (research randomizer) was used to randomly allocate each patient to one of three groups. To avoid bias, random sequencing was performed by one of the authors, who had not taken part in allocation or by the measurement statistician. Another author explained the study and introduced oral hygiene to the patients and their guardians according to random sequencing, which was provided in opaque, sealed envelopes during the first appointment. Also, the investigator who treated patients and collected data was blinded to all groups throughout the study. 

All the participants were referred to the department of Periodontology for plaque elimination, prior to orthodontic treatment. The educational goals in all of the educational interventions were consistent with the goals mentioned in the educational booklet of British Orthodontics Society (BOS) about oral hygiene (Table 1).^20^

**Table 1:** Learning objectives for oral hygiene education and components of fixed orthodontic appliance. Source: Thickett and Newton,^20^ 2006.

Learning objectives for oral hygiene education	Learning objectives for components of fixed appliance treatment
The equipment required for orthodontic oral care.	What are the components of oral fixed appliance therapy?
How do I use an orthodontic tooth brush?	The obligation for hygiene of the molar bands and molar tubes in every oral care episode.
How do I use an interspace brush?	What are the names of the two fixed appliance components to be checked at the end of daily oral care?
How do I use an ordinary tooth brush?	What should I do when my brackets are removed or my archwire is broken?
How do I use dental floss?	What precaution should I take in case of irritation caused by the brackets and molar tubes?
How do I use fluorinated mouthwash?	What are the basic rules when using elastics?
What are the names of the two fixed appliance components to be checked at the end of daily oral care?	What should I do in case of pain due to the appliance?
What kind of food do I have to avoid?	What is the duration of the therapy?
	What is the frequency of orthodontic appointments?
	Is it safe to attend sports activities?
	Is it safe to play any musical instrument?

Ninety consecutive fixed orthodontic appliance treatment candidates in compliance with the above-mentioned criteria were randomly assigned to three groups (n = 30 in each):

Control group (CG): The patients in the control group received standard printed educational material and were assisted with verbal information in accordance with BOS educational goals.^20^

**Figure 2:**
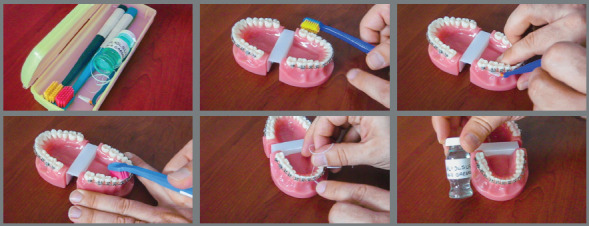
Sections from video-assisted education.

Study group 1 (S1): The patients in this study group received video-assisted information (Fig 2).

Study group 2 (S2): The patients in this study group received hands-on training. 

Both video and hands-on assisted training sessions covered a set of knowledge and skills on fixed appliance therapeutic devices and oral hygiene, as stated in the educational booklet of BOS (Table 1).^20^ The primary outcomes were the plaque and gingival indices.

The importance of eliminating dental plaque in oral health was emphasized and oral hygiene instructions were repeated by the same author during each appointment. Patients in all groups used the same kind of toothbrush and toothpaste throughout the study and were advised to brush their teeth at least three times a day for three minutes. The brushing habits of the patients were checked during each appointment and the orthodontic archwires were ligated with stainless steel wires. 

The video-assisted training consisted of two separate sessions, each one lasting five minutes. The first was about the treatment materials and the second about oral hygiene, in compliance with the BOS educational obligations. All the candidates were allowed to rewind and watch the video presentation until they reached all of the educational goals. The completion of the educational goals was documented through a written examination form filled by the candidates themselves. They all got the full score in the written examinations in both topics.

The hands-on training, consisting of two separate sessions, was performed by one of the authors. The first was about the treatment materials and the second about oral hygiene, in accordance with the BOS educational obligations. All of the patients were allowed to ask questions freely until they reached all of the educational goals. The completion of the goals was documented through a written examination form filled by the candidates themselves. They all got the full score in the written examinations in both topics. The time requirements for the three different educational interventions were recorded separately.

The adequacy of oral hygiene was documented through the Löe and Silness^21^ plaque and gingival indices, by one of the authors, who was blinded to the patients’ educational intervention group during the initial visit and the eighth week of the treatment. 

### STATISTICAL ANALYSIS

The statistical analysis was performed by using SPSS 25.0 (IBM Corporation, Armonk, NY, USA). The suitability of the data to normal distribution was assessed with the Shapiro-Wilk test (95% confidence intervals for means). The continuous variables were presented as mean ± standard deviation (SD); and categorical variables, in frequency and percentage. The group comparisons for continuous variables were performed using one-way analysis of variance (ANOVA). Also, Tukey HSD and Student *t*-test were used for *post-hoc* comparisons (α = 0.05). The group comparisons for categorical variables were performed using a chi-square test. The correlation between variables was analyzed using the Pearson correlation coefficient, and *p* values lower than 0.05 were accepted as significant in all the tests. 

## RESULTS

All of the patients in the control and study groups were comparable regarding age, gender and initial plaque and gingival indices values (*p*> 0.05) (Table 2). The time duration for standard educational intervention was 13.5 ± 2.2 min, while video-assisted education took 38.7 ± 8.0 min and hands-on training was 25.2 ± 6.5 min. These time durations consisted of the education time needed for both fixed appliance components and oral hygiene education. The mean total time duration for the educational intervention was found to be significantly shorter in the control group when compared with those of both study groups (*p*< 0.001) (Table 3). The mean time for video-assisted education was significantly longer than that of the hands-on training group (*p*< 0.001) (Table 4).

**Table 2:** Patient demographics, baseline characteristics and initial periodontal health conditions (n=30 per group).

Variables	CG	S1	S2	P
Age (Years)	15.5±3.1	14.0±2.3	14.7±2.3	0.11^†^
<18 years (n,%)	26 (87)	29 (97)	28 (93)	
≥18 years (n,%)	4 (13)	1 (3)	2 (7)	
Gender				0.35^‡^
Male (n,%)	7 (23)	11 (37)	12 (40)	
Female (n,%)	23 (77)	19 (72)	18 (60)	
Initial PI	0.49±0.34	0.39±0.37	0.50±0.26	0.39^†^
Initial GI	0.49±0.30	0.38±0.26	0.45±0.30	0.28^†^

CG, Control Group (Standard education); S1, Study Group 1 (Video-assisted education); S2, Study Group 2 (Hands-on training) ; PI, Plaque Index; GI, Gingival Index; ^†^ One way analysis of variance, ^‡^ Kruskall-Wallis analysis of variance. 95% Confidence Interval for Mean, *p* < 0.05, significant difference.

**Table 3:** Comparison of mean PI, GI and instruction times of different educational methods (n=30 per group).

Variable	Group	Mean± SD	df	F	P	Estimated effect size
Initial PI	CG	0.49±0.34^A^	2	0.96	0.39^†^	0.02
	S1	0.40±0.37^A^				
	S2	0.51±0.26^A^				
Eighth week PI	GC	0.97±0.40^B^	2	42.91	< 0.001^†^	0.5
	S1	0.41±0.14^A^				
	S2	0.41±0.21^A^				
Initial GI	GC	0.50±0.30^A^	2	1.30	0.28^†^	0.03
	S1	0.38±0.26^A^				
	S2	0.44±0.30^A^				
Eighth week GI	GC	1.19±0.20^B^	2	122.29	< 0.001^†^	0.74
	S1	0.36±0.26^A^				
	S2	0.45±0.20^A^				
Total time for education (min)	GC	13.53±2.19^A^	2	128.17	< 0.001^†^	0.75
	S1	25.17±6.47^B^				
	S2	38.70±8.04^C^				

CG, Control Group (Standard education); S1, Study Group 1 (Video-assisted education); S2, Study Group 2 (Hands-on training); PI, Plaque Index; GI, Gingival Index; SD, Standard deviation; df, numerator degrees of freedom. Different superscript letters denote significant differences according to Tukey HSD post-hoc test. ^†^One-way analysis of variance. 95% Confidence Interval for Mean, *p* < 0.05, significant difference.

**Table 4:** The comparison of the study groups for duration of education, plaque index and gingival index (n=30 per group).

Variables	S1		S2		F	df	P
	Mean± SD	SE	Mean± SD	SE			
Initial PI	0.40±0.37	0.07	0.51±0.26	0.05	0.07	58	0.188^†^
Eighth week PI	0.41±0.14	0.03	0.41±0.21	0.04	2.75	58	0.972^†^
Initial GI	0.38±0.26	0.05	0.44±0.30	0.05	0.15	58	0.336^†^
Eighth week GI	0.36±0.26	0.05	0.45±0.20	0.04	0.96	58	0.171^†^
Total time for education (min)	25.17±6.47	1.18	38.70±8.04	1.47	2.48	58	<0.001^†^

S1, Study Group 1 (Video-assisted education); S2, Study Group 2 (Hands-on training); PI, Plaque Index; GI, Gingival Index; SD, Standard deviation; SE, Standard Error; ^†^Student t-test; 95% Confidence Interval for Mean, *p*< 0.05, significant difference.

The two study groups did not show any significant intergroup or in-group deterioration regarding plaque and gingival indices at the initial eighth week examinations (*p*> 0.05) (Table 4). However, the eighth week plaque and gingival indices in the control group patients significantly deteriorated when compared with the initial index values (*p*< 0.001) (Table 3). The eighth week plaque and gingival indices of the control group were also significantly poorer than those of the two study groups (*p*< 0.001) (Table 3).

The time duration spent on patient education was found to be inversely and strongly correlated with the preservation rate of the plaque and gingival indices. The longer the duration of education - including both fixed appliance treatment components and oral hygiene - the better the preservation rate of the plaque index (r = -0.535, *p* < 0.001) and gingival index (r = -0.561, *p* < 0.001) in the eighth week of treatment.

## DISCUSSION

In this study, plaque and gingival indices of patients receiving different educational methods were compared in the eighth week of treatment. The plaque index values of the three groups was significantly affected by different educational methods (*p*< 0.05). Therefore, the first null hypothesis was accepted. In addition, the training time of the video-assisted and hands-on methods were examined separately. The video training method took less time than the hands-on method (*p*< 0.05). Therefore, the second null hypothesis was rejected.

The present study investigated the success and clinical efficacy of different educational interventions on oral hygiene motivation in individuals undergoing fixed orthodontic treatment. This is because fixed appliance treatment is known for its deleterious effects, such as gingivitis, white spots, decalcification and cavity formation unless oral hygiene is maintained by the patients themselves.^22^ ^-^ ^25^ The institution of oral hygiene before the onset of orthodontic treatment is recommended as an effective means against development of the above-mentioned complications.^17^ As it becomes more difficult to sustain oral hygiene following the application of therapeutic appliances,^26^ the educational intervention for oral hygiene behaviour and the orthodontic treatment materials take priority in the beginning of the treatment.^17^ The only known and successful way for achieving desired oral hygiene is both educating the patient prior to the treatment and building rapport between doctor and patient during the long-term treatment.^8^

The effectiveness of different methods in improving oral hygiene compliance of patients undergoing fixed orthodontic treatment to minimize these harmful effects has been investigated in previous studies. Since orthodontists worry that patients’ compliance will decrease during the 4-6 week appointment intervals of the treatment,^19^ the patients were motivated by a number of reminder messages or applications (text, WhatsApp, WeChat) that emphasized the importance of good oral hygiene, and the effectiveness of these reminders was investigated. In all of these studies, it was stated that the use of reminders in dentistry improved the patient’s out-clinical management, regular attendance of appointments, positive behaviour changes and educational factors.^10^ ^,^ ^14^ ^,^ ^16^ ^,^ ^27^ However, many smartphones have the ability to block messages and they can be easily blocked if they cause annoyance to patients.^19^ In addition, it has been emphasized in studies that plaque disclosing tablets increase oral hygiene motivation through visualization of plaque accumulation.^11^ ^,^ ^12^ Although it is easily accessible and practiced in orthodontic clinics, its use may be limited due to its price, especially in developing and underdeveloped countries.^19^

Although the above-mentioned oral hygiene motivations were less time-consuming per patient, we tested educational interventions, which are more widely accepted by orthodontic patients and have become a long-term habit. Patient education on oral hygiene behaviour was found to be successful either through verbal, written or visual educational interventions.^8^ ^-^ ^11^ These techniques have been proven to be more effective when used in combination rather than independently.^8^ It is also known that hands-on training together with verbal and written educational means improves the success rates in plaque and gingival indices preservation during orthodontic treatment.^15^

The main purpose of the present study was to compare the time duration spent on educational interventions through three different modes of information transfer, and to evaluate its relation with positive outcomes in plaque and gingival indices. In addition, the effectiveness of the standard, video-assisted and hands-on education techniques were also compared. The study showed that both study groups succeeded in maintaining oral hygiene without any significant difference between the preservation rates of plaque and gingival indices (*p*> 0.05). Although both video and hands-on training assisted educations took a considerable amount of time for the orthodontist, the time duration of hands-on education was found to be significantly shorter than that of video-assisted education (*p*< 0.001). This may provide the orthodontist two options to choose from: either a time-consuming option (video-assisted) or an effort consuming (hands-on assisted) method, depending on their constraints. On the other hand, the control group, which received standard educational intervention, failed to preserve oral hygiene, at least at the eighth week of treatment.

Although it is time and effort-consuming, the video and hands-on assisted educational techniques are seemingly more desirable for preserving plaque and gingival indices (*p*< 0.001), hence preventing various complications from the treatment. The oral hygiene education covered topics such as the names of the therapeutic appliances, the maintenance of these materials and the effective use of oral hygiene tools. Patients had to remember in full and properly apply this information after the educational intervention. In addition, they had to overcome their powerful and negative urges and implicit behavioural retreat from oral hygiene. The motivation of the orthodontist in implementing oral hygiene through educational intervention is important for the patients to understand and behave according with oral hygiene precautions. Repetition of the topics related to oral hygiene during successive appointments is also recommended.^28^

The first limitation of the study was the Hawthorne effect,^9^ which resulted from the inability to blind patients. It was not possible to eliminate this because of the consent form obtained from patients and their parents for participation in the study. The second limitation was that the follow-up period of the patients was limited to eight weeks and long-term follow-up was not managed. However, this lack can be considered normal as we focus on training durations rather than the success of oral hygiene motivation in planning our study.

## CONCLUSION

In a couple of previous studies, the provision of oral hygiene behaviour education by orthodontists or oral hygienists was emphasized.^9^ ^-^ ^11^ ^,^ ^13^ ^,^ ^14^ However, the time and personnel constraints were not addressed in those studies. We found that standard verbal and written educational interventions are better supported by either video or hands-on assisted educational programs depending on the constraints of the orthodontist. The longer the educational intervention is, the better the preservation of the plaque and gingival indices. Presumably, the orthodontists may help their patients with a safer therapeutic approach by providing them with a longer duration of education either by making them view video recordings in the waiting room or letting them perform hands-on training about proper orthodontic oral hygiene and fixed appliance treatment, under the supervision of a dental hygienist.
